# Low-Intensity Pulsed Ultrasound Protects Retinal Ganglion Cell From Optic Nerve Injury Induced Apoptosis via Yes Associated Protein

**DOI:** 10.3389/fncel.2018.00160

**Published:** 2018-06-13

**Authors:** Jia-Xing Zhou, Yun-Jia Liu, Xi Chen, Xi Zhang, Jie Xu, Ke Yang, Dong Wang, Sen Lin, Jian Ye

**Affiliations:** ^1^Department of Ophthalmology, Research Institute of Surgery, Daping Hospital, Army Medical University, Chongqing, China; ^2^Chongqing Engineering Technical Center Stem Cell Therapy, Children’s Hospital of Chongqing Medical University, Chongqing, China; ^3^Department of Ultrasound, The First Affiliated Hospital of Chongqing Medical University, Chongqing, China; ^4^Chongqing Key Laboratory of Ultrasound Molecular Imaging, The Second Affiliated Hospital of Chongqing Medical University, Chongqing, China

**Keywords:** LIPUS, retina, RGC, YAP, apoptosis

## Abstract

**Background:** Low-intensity pulsed ultrasound (LIPUS) has been used in clinical studies. But little is known about its effects on the central nervous system (CNS), or its mechanism of action. Retinal ganglion cells (RGCs) are CNS neuronal cells that can be utilized as a classic model system to evaluate outcomes of LIPUS protection from external trauma-induced retinal injury. In this study, we aim to: (1) determine the pulse energy and the capability of LIPUS in RGC viability, (2) ascertain the protective role of LIPUS in optic nerve (ON) crush-induced retinal injury, and 3) explore the cellular mechanisms of RGC apoptosis prevention by LIPUS.

**Methods:** An ON crush model was set up to induce RGC death. LIPUS was used to treat mice eyes daily, and the retina samples were dissected for immunostaining and Western blot. The expression of yes-associated protein (YAP) and apoptosis-related proteins was detected by immunostaining and Western blot *in vitro* and in *vivo*. Apoptosis of RGCs was evaluated by TUNEL staining, the survival of RGCs and retained axons were labeled by Fluoro-gold and Tuj1 antibody, respectively. Rotenone was used to set up an *in vitro* cellular degenerative model and siYAP was used to interfering the expression of YAP to detect the LIPUS protective function.

**Results:** LIPUS protected RGC from loss and apoptosis *in vivo* and *in vitro*. The ratio of cleaved/pro-caspase3 also decreased significantly under LIPUS treatment. As a cellular mechanical sensor, YAP expression increased and YAP translocated to nucleus in LIPUS stimulation group, however, phospho-YAP was found to be decreased. When YAP was inhibited, the LIPUS could not protect RGC from caspase3-dependent apoptosis.

**Conclusion:** LIPUS prevented RGCs from apoptosis in an ON crush model and *in vitro* cellular degenerative model, which indicates a potential treatment for further traumatic ON injury. The mechanism of protection is dependent on YAP activation and correlated with caspase-3 signaling.

## Introduction

Traumatic injury-induced optic nerve (ON) degeneration and glaucoma-triggered possessive retinal ganglion cell (RGC) death are the main challenges of vision loss. The RGCs convey visual signals from the retina along axons to specific brain regions. Axon injury and collateral degeneration result in RGC soma and dendrite atrophy and finally RGC death. In human, RGC deterioration accompanies glaucoma neuropathy or cell death quickly after traumatic ON injury. Regardless of injury progression, it is urgent and necessary to prevent RGC from apoptosis to preserve the basic structure of the retina and further cell death.

The molecular mechanisms of RGCs apoptosis in glaucoma and traumatic optic neuropathy are complex. It has been reported that mitogen-activated protein kinases (Katome et al., 2013), mitochondrial dysfunction (Kim et al., 2015), p53 (Wilson et al., 2013), Fas ligand (Krishnan et al., 2016), and caspases (Li et al., 2009) are in association with RGCs apoptosis. However, clinical treatment is far more complicated. The current clinical protective methods primarily suggested surgical optic canal decompression (Kong et al., 2011; Vaitheeswaran et al., 2014). Besides surgical method, noninvasive methods have shed some lights on the treatment of axon injury and neuronal protection. Ultrasound is commonly used as a clinical method to obtain an inner body image for diagnosis. In peripheral nervous system (PNS), noninvasive low-intensity ultrasound was used to sustain neuronal soma survive and to promote peripheral nerve regeneration with functional recovery by stimulating brain-derived nerve growth factor (BDNF) release (Ni et al., 2017). *In vitro* study demonstrated that nerve growth factor (NGF) combined with low-intensity pulsed ultrasound (LIPUS) stimulation may have some effects on PC12 cell neurite outgrowth (Zhao et al., 2016). Moreover, transected inferior alveolar nerve with LIPUS facilitated morphological and functional regeneration which suggested the potentiation of LIPUS as a novel therapy for PNS injury (Sato et al., 2016). Even without growth factor combination, the ultrasound was able to accelerate autograft nerve regeneration at energy of 250 mW/cm^2^ (Jiang et al., 2016). Not only in PNS, LIPUS also takes some functions in central nervous system (CNS) recovery. In an acute spinal cord injury (SCI) model, the combination of ultrasound irradiation and NGF/poly lactic-co-glycolic acid (PLGA) nanobubbles holds some promising effect on SCI (Song et al., 2017). Moreover, it was indicated transcranial focused ultrasound could modulate the activity of primary somatosensory cortex in humans (Legon et al., 2014). Due to the noninvasive effect of ultrasound, the hippocampus and motor cortex as well as retinas could be stimulated in intact mice (Tufail et al., 2010; Menz et al., 2013). As for a short exposure time at low intensity, ultrasound-triggered neuronal activity is thought partially stem from the mechanical pressure effect, but not tissue heating. However, the intracellular mechanism still needs to be elucidated in the retina (Tufail et al., 2010).

In this study, to verify the LIPUS neural protective hypothesis, we utilized mice ON crush model and *in vitro* RGC degenerative model, aimed to modulate the retina with LIPUS to explore the potential effects of LIPUS on RGC survival and corresponding mechanism.

## Materials and Methods

### Ultrasound Energy Measurement

The ultrasonic therapeutic apparatus was designed and manufactured by medical ultrasound engineering institute of Chongqing Medical University, China (Ultrasonic probe diameter: 2.5 cm, acoustic frequency 1 MHz, duty cycle: 20%, pulsed repetition frequency 1 KHz). We utilized ultrasound power meter (UPM-DT-1AV, Ohmic Instruments, United States) to measure the ultrasound output energy and consistent level. Briefly, the ultrasonic probe was gripped by the gripper and the metal part of the generator was placed under water above the detect cone. We chose different power grades to measure the acoustic intensities [Grade 1 (minimum), Grade 2, Grade 3, Grade 5, Grade 10, Grade 15, and Grade 20 (max)]. Each grade was repeated for six times, independently, and the outputs of energy were showed as Joule (J, mean ± SD).

### RGC and Cortical Neuron Isolation and Purification

Retinal ganglion cells were isolated from postnatal (P5) wild-type mice [C57BL/6J, purchased from Animal Center of Army Medical University (AMU)] and cortical neurons were from E18 mice (Barres et al., 1988; Lin et al., 2012; Wang et al., 2017). Revised from Goldberg’s and Winzeler’s protocol (Goldberg et al., 2002; Winzeler and Wang, 2013), briefly, retinas were dissected from the eye cup of P5 mice on ice. After Hank’s balanced salt solution (HBSS) rinse, the retinas were digested by TRPLE solution (Gibco, 12605010, United States) at 37°C for 15 min, which was terminated by rinsing the cells with increasing concentration of ovomucoid (20–40 mg/ml). Re-suspended the retinal cells in panning buffer containing insulin (5 μg/ml) and then incubated with an RGC-specific Thy-1 antibody (LS-C14009, LifeSpan, United States). The Thy-1 plate was rinsed multiple times with Earle’s balanced salt solution (EBSS). RGCs were released from the panning dish with trypsin (Sigma–Aldrich, United States) which was terminated by Neurobasal medium (12349-015, Gibco by Life Technologies, United States) plus 33% fetal bovine serum (Gibco by Life Technologies, United States). RGCs were plated at a high density of 10^6^ cells/ml on poly-D-lysine (10 μl/ml, Sigma–Aldrich, United States)-coated cell culture plates (Sigma, Shanghai, China). The RGCs were kept in culture at 37°C with 5% CO_2_ for 6 days (d). The growth culture medium is formulated from Neurobasal medium mixed in equal amounts with DMEM high-glucose medium (31966, Gibco, United States) with addition of penicillin (1000 units/ml), streptomycin (1000 μg/ml), insulin (0.5 μg/ml), sodium pyruvate (1.5 mM), 3,3^′^,5-triiodo-L-thyronine (40 ng/ml), L-glutamine (3.7 mM), and B27 supplement (1×, Gibco, United States). Primary mouse cortical neurons were derived from E18 wild-type or indicated mutant embryos (Wang et al., 2017). Briefly, cortical tissues were dissected from embryos of both sexes and digested in HBSS containing papain (Worthington, LS003126) at 37°C for 15 min. Neurons were plated on poly-L-lysine-coated coverslips or dishes (100,000 cells/well for 24-well dish, 600,000 cells/ well for 6-well dish). Cultures were maintained with glia-conditioned media in Neurobasal, 2% B-27 supplement, 2 mM Glutamax, and 1% horse serum (Thermo Fisher Scientific Inc., Rockford, IL, United States) in a humidified incubator at 37°C with 5% CO_2_. This study was carried out in accordance with the recommendations of ARRIVE guidelines in accordance with the National Institutes of Health guide for the care and use of Laboratory animals. The protocol was approved by the Animal Ethics Committee of AMU.

### Transfection of siRNA to RGCs

Yes-associated protein (YAP) siRNA is a pool of 3-target-specific 19–25 nt siRNAs designed and manufactured by Santa Cruz Biotechnology Inc. (Santa Cruz, TX, United States) to knock down YAP gene expression. Briefly, RGCs (days *in vitro*, DIV4) were seeded at 1 × 10^5^ cells/well in neural basal medium (Life Technologies, United States) in 24-well plates the day prior to transfection. A mixture of siRNA duplex (1.5 μl/well) and siRNA transfection reagent (lipo3000, 1.5 μl/well, Life Technologies, United States) was formed in transfection medium (47 μl/well). Complexes were added to wells containing cells and incubated for 2 h at 37°C in 0.2 ml of siRNA transfection medium. All experiments with silenced cells were performed 48 h post-transfection in triplicate, and they were repeated three times. A similar procedure was used with scrambled siRNA (control siRNA, sc-37007) to rule out the occurrence of off-target effects. The data shown are from representative experiments.

### Ultrasound Dosage-Dependent Cell Viability Assay

Primary cortical neuron and primary RGCs were used to measure the suitable energy level of LIPUS. The cells were seeded in a medium of Neurobasal medium in 5% CO_2_ condition in 96-well plate in a density of 1 × 10^5^/ml. LIPUS output energy was measured in a time-dosage assay by CCK-8 kit (Dojindo, Japan) according to manufacturer’s protocol. Briefly, after cell plating for 4 h, 10 μl CCK-8 solution was added in to each well to culture for 4 h. Under 450–490 nm wavelength detection and 600–650 nm reference wavelength, optic density (OD) values of LIPUS-treated primary cortical neuron and RGC cells could be read and recorded by Biotek Elx800 (Winooski, VT, United States). Therefore, by energy-viability calculation we could obtain an optimal time-dosage assay, which provides suitable basic data for further *in vitro* LIPUS cell viability research.

### Optic Nerve Crush Surgical Procedure

The mice ON crush model is considered as a classic tool for studying CNS regeneration, which was performed as we did before (Lin et al., 2012). Eight-to-ten-week old adult WT C57BL/6J male mice, weighed about 24g, were purchased from Animal Center of AMU, and anesthetized with intraperitoneal injection of pelltobarbitalum natricum in saline (70–80 mg/kg). A small incision was made in one eye in the superior posterior area of the conjunctiva, eye muscles were gently moved to expose the ON. The nerve was crushed with extra-fine self-closing forceps at 1.5 mm behind the globe for 9 s without damaging the retinal vessels or blood supply (Lin et al., 2012; Li et al., 2017). All procedures were performed aseptically and on the left eye, with the right eye serving as a sham operated control. All experiments met the criteria outlined by the Institutional Animal Care and Use Committee of AMU, and were performed according to the guidelines in The Handbook of the Laboratory Animal Center, AMU. The Animal Ethics Committee of AMU approved all experimental procedures used in the present study. All mice were housed on a 12 h light/dark schedule with water and food available *ad libitum*. Neonatal C57BL/6 mice were used to make primary RGC cultures.

### Fluoro-Gold (FG) Retrograde Labeling

Wild-type adult male mice were anesthetized and placed in a stereotactic apparatus (Stoelting, Kiel, WI, United States) as we did before (Lin et al., 2012). The skull was exposed and cleaned with 3% hydrogen peroxide. The accurate location of superior colliculus was conducted followed mice brain atlas (The Mouse Brain in Stereotaxic Coordinates, Academic Press, New York, 2001). A hole of 1 mm in diameter was drilled in the skull (4.4 mm posterior and 0.06 mm lateral to the bregma), and a 26-gauge stainless steel cannula was inserted for infusion of fluorochrome, hydroxystilbamidine (FG; Biotium, Inc., Hayward, CA, United States) at a speed of 1 μl/10 min. One week before ON lesion. One microliter of 2% FG was injected into the bilateral superior colliculus (1.2 mm deep from the skull). To analyze the survival number of RGCs in whole retinas labeled with FG at 7-d post-ON crush (ONC7d), the golden dots (survived RGCs) were counted using FIJI software (NIH, United States).

### Spectralis Optical Coherence Tomography (OCT)

Before and after ON crush modeling, the retinas from the ONC and ONC+LIPUS groups were analyzed by using *S*-optical coherence tomography (*S*-OCT) at different ON degeneration stages, such as before the lesion (baseline), 1 3, 7, and 14 d after the injury. At the end of the study (14 d) animals were euthanized and the retinas dissected for further analysis. Briefly, animals were anesthetized by pelltobarbitalum natricum (70–80 mg/kg) i.p., a drop of tropicamide (1%; Alcon-Cusi, S.A., Barcelona, Spain) was instilled in both eyes to induce mydriasis. Eyes were kept hydrated with artificial tears, a custom-made contact permeable lens (3.2-mm PMMA lens, cornea surface R1.7 mm, outer surface R1.8 mm, central thickness about 0.3 mm) was placed on the cornea to maintain corneal hydration and clarity. The retinas were imaged according to manufacturer’s instructions (Spectralis; Heidelberg Engineering, Heidelberg, Germany). For the adaption of mice eye, a commercially available 27 dpt double aspheric fundus lens (Heidelberg Engineering, Heidelberg, Germany) was mounted in front of the camera unit. Imaging was performed with a Heidelberg image capture module (HRA2 Spectralis Family Acquisition 6.0.11.0, HRA spectralis Viewing Module 6.0.9.0; Heidelberg Engineering, Heidelberg, Germany) as described. Retinas were scanned with a raster pattern of 31 equally spaced horizontal B-scans spanning the central retina (3000-μm length). In four central sections per animal, the retinal nerve fiber layer (RNFL) and retinal thickness were manually measured at a range of 500 (center) to 1500 (periphery) μm from the ON. The average retinal thickness was calculated automatically by the software. Six individual eyes from either crush or crush+LIPUS groups were calculated separately, and the data were analyzed by SPSS 18.0 software.

### Preparation of the Whole-Mount Retina and Tissue-Immunostaining

Animals were sacrificed by over-dosage CO_2_ inhalation. After 0.9% saline perfusion, 4% paraformaldehyde (PFA) was used to fix retina as we did before (Lin et al., 2012). Retina cups were carefully dissected, four cuts were made to enable the tissue to lie flat, then post-fixed it in 4% PFA for 1 h at 4°C. After PBST rinsing, we used 3% triton-x 100 solution to permeabilize the retinal tissue over night at 4°C. The next day, retinas were rinsed with PBST three times and blocked with 3% donkey serum for 2 h at room temperature (RT). Add Tuj1 antibody (1:500, mouse monoclonal; Covance, Cat.MMS435P) to incubate the whole mount retinas for 2 d at 4°C. This step was followed by incubation with appropriate fluorescently labeled secondary antibody (1:400, anti-mouse Alexa Fluor 488, molecular probes, Thermo Fisher Scientific Inc., Rockford, IL, United States) over night at 4°C. The images were taken by SP-8 confocal microscope (Leica, Germany). FIJI software was used to count βIII-tubulin immune-positive cells from eight fluorescent images per retina (200×, SP-8, Leica, Germany), four at 1 mm and four at 2 mm apart from the ON head, respectively, the number was averaged to estimate overall RGC survival per square millimeter. The ODs of axon bundles in area of interest (AOI) were calculated by FIJI software. Briefly, the Tuj1-labeled ON bundles in whole mount retina were transferred to 8-bit image. Adjust the threshold automatically to get a high contrast image and analyze the mean OD of the axon bundles.

### Ultrasound Treatment of Eye

After surgery simultaneously, the LIPUS treatment was started to stimulate the retina and ONs with an ultrasonic therapeutic apparatus (designed and manufactured by medical ultrasound engineering institute of Chongqing Medical University, China). Ultrasonic probe diameter 1.3 cm, acoustic frequency 1 MHz, duty cycle 20%, and pulsed repetition frequency 1 kHz. Acoustic intensity (spatial average temporal average, SATA): low-dose group (Grade 1), mid-dose group (Grade 2), and high-dose group (Grade 5). The ultrasonic therapeutic apparatus sets 1–20 different intensity grades. Grade 1, Grade 2, and Grade 5 represent the intensities of 50, 150, and 450 mW/cm^2^ ultrasound output according to the manufacturer’s manual. The energy can be calculated by the formula: intensity (mW/cm^2^)/1000× (15, 30, 45, 60, 90, 120, 180, or 240 s)/3600 × 3.6 × 10^6^ (kW/h). Total treatment time: 10 min each time from 1 to 7 d post-crush.

### Immunostaining

After 0.9 and 4% PFA perfusion, fixation, and post-fixation, tissues underwent immunostaining after cryosection (10 μm, embedded in OCT Tissue Tek Medium, Sakura Finetek). After blocking with the appropriate sera, sections were incubated overnight at 4°C with primary antibodies. The next day, sections were washed three times, incubated with appropriate fluorescent secondary antibodies (1:400, molecular probes, Thermo Fisher Scientific Inc., Rockford, IL, United States) 1 h at RT. After DAPI staining, the slides were immediately mounted. Images were taken by SP-8 confocal microscope (Leica, Germany). The primary antibodies used in this work are Tuj1 (1:500, mouse monoclonal; Covance, Cat. MMS435P), SMI-32 (1:400, Abcam, Cat. Ab8135), YAP, and Phosphorylated YAP**^Ser127^** (p-YAP^Ser127^) (1:100, Cell Signaling Technology, United States, Cat. 13008).

### Immunoblotting

For freshly dissected retinal tissue and primary cultured RGCs, RIPA (Thermo, 89900) was used to obtain the lysate as we did before (Lin et al., 2012). Protease inhibitor cocktail (118836153001; Roche Diagnostics, Indianapolis, IA, United States) was used to inhibit protein degradation. The lysate was then separated by centrifugation at a speed of 12,000 × *g* at 4°C for 15 min. The supernatant was collected and the protein concentration was measured using a bicinchoninic acid protein assay (Pierce, Rockford, IL, United States). Forty micrograms of protein samples was loaded into SDS-polyacrylamide gels. Proteins were then transferred to polyvinylidene difluoride membranes (Millipore Corporation, Bedford, MA, United States) using 100 V current for 1.5–2 h. The blots were then first washed with Tris-buffered saline and Tween (TBS-T; 50 mmol/l Tris pH 7.4, 150 mmol/l NaCl, and 0.1% Tween), followed by blocking in 5% nonfat milk-TBS-T overnight at 4°C. Antibodies recognizing YAP1 (1:500, Rabbit Monoclonal, Cell Signaling Technology, 14074s), p-YAP^Ser127^ (1:500, Rabbit Monoclonal, Abcam, ab56701), caspase-3/cleaved caspase-3, and GAPDH (1:5000, Mouse Monoclonal, Abcam, ab9484) were made up in a solution of 5% milk in TBS-T, and used overnight at 4°C, followed by three washes with TBS-T and incubation with horseradish peroxidase-conjugated anti-rabbit or anti-mouse IgG secondary antibodies (Santa Cruz Biotechnology Inc., Santa Cruz, CA, United States) in TBS-T for 1.5 h at RT. Commercial chemiluminescent detection system (Super-Signal^®^ West Pico Chemiluminescent Substrate Detection System; Thermo Fisher Scientific Inc., Rockford, IL, United States) was used to get immunoblotting results, images were taken by gel image system (Aplegen, Gel Company, Inc., San Francisco, CA, United States).

### TUNEL

Apoptotic cell death was detected in the retinal sections following the protocol of TUNEL kit (Sigma, Shanghai, China, 11684795910 and 11684817910). Briefly, retina sections were fixed by PFA (4%) for 15 min at 4°C, after PBS wash and citrate/triton buffer (0.1%) permeabilization, mixed TUNEL detect solution was added to the sections and then the samples were incubated for 50 min at 37°C in a dark and humid condition. After PBS wash the sections were mounted and the images were taken by SP-8 confocal microscope (Leica, Germany). TUNEL-positive signals were counted by using FIJI software, randomly, six high-power frames were chosen from each section to obtain the average number of TUNEL-positive cells, three sections were selected from each animal.

### Statistical Analysis

Data in LIPUS intensities and OCT measurement are presented as mean ± SD. Other data sets from at least three independent experiments were presented as mean ± SEM. Independent group comparisons were analyzed by using one-way ANOVA. *P*-value of less than 0.05 (*P* < 0.05) was considered to be statistically significant. Each experiment was performed independently more than three times.

## Results

### Ultrasound Energy Sustained Stable at Grade 1 and Grade 2 But Unstable at Grade 3, Grade 5, Grade 10, Grade 15, and Grade 20

Prior to the biological experiments, accurate energy extent of ultrasonic apparatus was measured by ultrasound power meter (UPM-DT-1AV). Based on the instruction of the user’s guide, the energy power output ranged from Grade 1 to Grade 20 are listed in **Table 1**. Referred to the literature, stimulation in CNS ranged from 30 mW/cm^2^ to 100 mW/cm^2^ below the recommended limit for clinical diagnostic imaging application (O’Brien, 2007; Legon et al., 2014). Correspondingly, we adopt low power LIPUS of Grade 1, Grade 2, Grade 3, Grade 5 (represent the energy of 50, 130, 260, and 450 mW/cm^2^ according to the manufacturer’s manual) and high power levels of Grade 10, Grade 15, and Grade 20 (represent the high power of 1.12, 2.68, and 4.76 mW/cm^2^ as the manual indicated) to measure the actual energy output. However, the energy of LIPUS is not as persistent stable as is showed in manufacture’s manual. As we measured, the energy of Grade 1 ranged from 40.5 ± 1.857 to 40.3 ± 0.919 mW/cm^2^ from 2nd to 10th-min duration of measurement, Grade 2 ranged from 123 ± 2.781 to 110.667 ± 3.138 mW/cm^2^, Grade 3 ranged from 230.333 ± 2.985 to 191 ± 1.915 mW/cm^2^, and Grade 5 ranged from 427.167 ± 8.345 to 354.647 ± 10.467 mW/cm^2^. As for a high power level, Grade 10, Grade 15, and Grade 20 can be measured from 1151.833 ± 9.393 to 416.333 ± 17.175 mW/cm^2^, 2441.833 ± 65.393 to 914.167 ± 46.064 mW/cm^2^, and 4400.5 ± 84.219 to 1348.5 ± 55.253 mW/cm^2^. However, the energy of the three typical high grades unstable after 2 min-test and attenuated gradually (**Table 1** and Supplementary Figure S1A).

**Table 1:** Intensities of LIPUS in different output grades on 2nd, 4th, 6th, 8th, and 10th-min treatment duration (*n* = 6).

	Grade 1 (mW/cm^2^)			Grade 2 (mW/cm^2^)			Grade 3 (mW/cm^2^)			Grade 5 (mW/cm^2^)			Grade 10 (mW/cm^2^)			Grade 15 (mW/cm^2^)			Grade 20 (mW/cm^2^)		
	Mean	*SD*	*N*	Mean	*SD*	*N*	Mean	*SD*	*N*	Mean	*SD*	*N*	Mean	*SD*	*N*	Mean	*SD*	*N*	Mean	*SD*	*N*
2nd min	40.500	1.857417	6	123.000	2.780887	6	230.3333	2.985148	6	427.167	8.3451	6	1151.833	9.392964	6	2441.833	65.35871	6	4400.500	84.21946	6
	*P* = 0.9404, vs. 10th min			*P* = 0.0687, vs. 10th min			*P* < 0.001, vs. 10th min			*P* < 0.001, vs. 10th min			*P* < 0.0001, vs. 10th min			*P* < 0.0001, vs. 10th min			*P* < 0.0001, vs. 10th min		
4th min	39.16667	2.151227	6	118.1667	3.18765	6	220.1667	2.120011	6	420.545	7.7445	6	991.8333	35.73459	6	2067.500	26.67302	6	3410.833	79.76608	6
6th min	38.66667	1.837873	6	118.1667	4.222295	6	210.500	2.741654	6	402.457	8.4516	6	812.500	15.59006	6	1542.167	96.15107	6	2443.167	52.15197	6
8th min	42.16667	1.81506	6	110.6667	3.602468	6	200.6667	4.977728	6	387.186	6.4874	6	631.000	15.69501	6	1243.500	33.39735	6	1799.333	66.48592	6
10th min	40.33333	0.9189366	6	110.6667	3.137586	6	191.000	1.914854	6	354.647	10.467	6	416.3333	17.17492	6	914.1667	46.06403	6	1348.500	55.25321	6

### The Low Grade LIPUS Has a Beneficial Effect on Cell Viability

**FIGURE 1:**
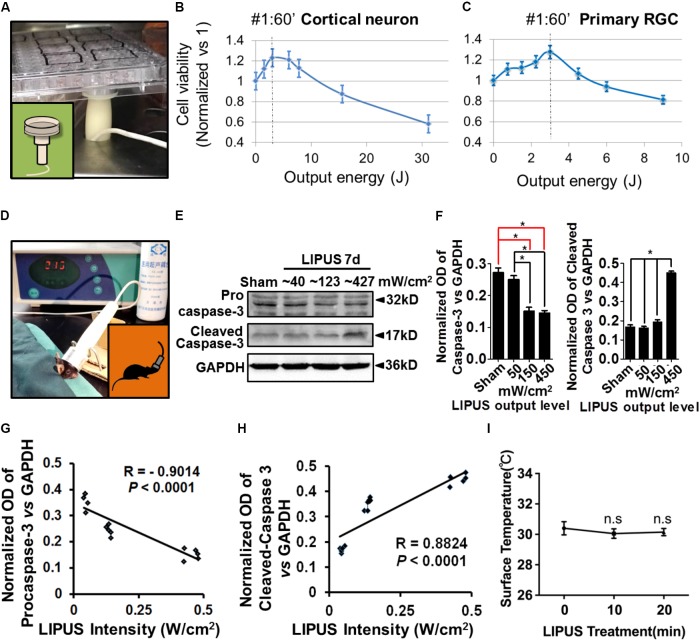
Low intensity of ultrasound has beneficial effect on cell viability *in vivo* and *in vitro.* **(A)** An example of LIPUS treatment to cells, *in vitro*. **(B)** The primary cortical neuron viability and LIPUS output energy assay (*n* = 6). **(C)** The primary RGC viability and LIPUS output energy assay (*n* = 6). **(D)** An example of LIPUS treatment on mice. **(E)** The LIPUS dose-dependent caspase-3 activation assay. Expression of procaspase-3 and cleaved-caspase-3 in different grades of LIPUS-treated groups at 7 d *in vivo*. **(F)** The normalized OD of procaspase-3 and cleaved-caspase-3 vs. GAPDH in different grades of LIPUS. Grade-5 LIPUS triggered more cleaved-caspase-3 (^∗^*P* < 0.05, *n* = 4). **(G)** The normalized OD of caspase-3 was negatively correlated with LIPUS intensities (*R* = 0.–0.9014, *P* < 0.0001, *n* = 4). **(H)** Cleaved-caspase-3 was positively correlated with the intensities (*R* = 0.8824, *P* < 0.0001, *n* = 4). **(I)** No significant temperature difference between 10 and 20 min treatment (*P >* 0.05, *n* = 9).

Considering the ultrasound sensitivity differences between PNS and CNS, we screened different energy-dosage and time-windows of ultrasound to explore the suitable power thresholds. As the results showed, Grade 1 and Grade 2 ultrasound has beneficial effects on viability of primary cortical neurons and RGCs in 60 s, twice per day, but has detrimental effects when treated duration extended (**Figures 1A–C**). Cell viability (normalized vs. 1) can be calculated according to the formula: cell viability = (OD450 LIPUS treatment-OD450 blank)/(OD450 control-OD450 blank). As for retina stimulation, the ultrasound (Grade 1, Grade 2, and Grade 5) was used at a time-duration from 10 to 20 min. Grade 5 LIPUS caused increased cleaved caspase-3 which suggested apoptotic and detrimental effect to retinal tissues (**Figures 1D–F**, ^∗^*P* < 0.05, *n* = 4). The normalized OD of pro-caspase-3 was negatively correlated with LIPUS intensities (**Figure 1G**, *R* = 0.–0.9014, *P* < 0.0001, *n* = 4), while cleaved caspase-3 was positively correlated with the intensities (**Figure 1H**, *R* = 0.8824, *^∗^P* < 0.0001, *n* = 4). No significant change of temperature was found between 10- and 20-min treatment (**Figure 1I**, *P >* 0.05, *n* = 9).

### LIPUS Protected RGC From Loss and Axon Atrophy After Optic Nerve Crush

**FIGURE 2:**
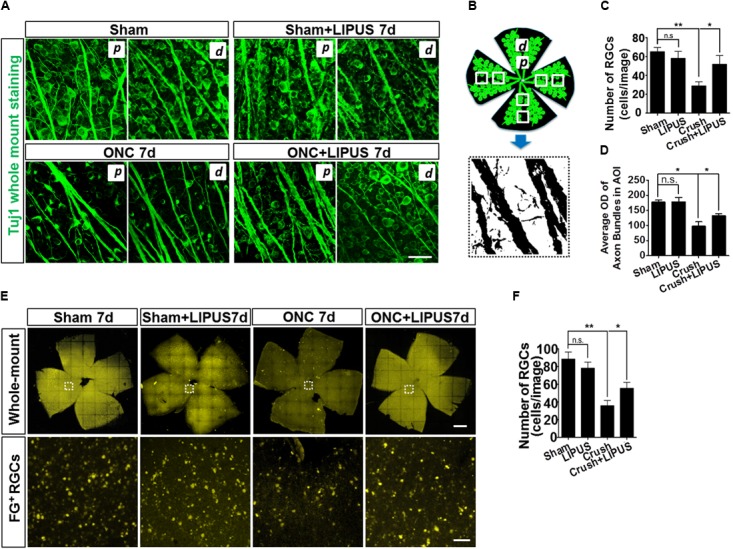
LIPUS protected retinal axon bundle from atrophy and RGC from loss. **(A)** Tuj1-immunopositive whole mount staining in sham, sham+LIPUS-7d, ONC-7d, and ONC+LIPUS-7d groups. Scale bar = 25 μm. *d*, distal region; *p*, proximal region. **(B)** The brief method to quantify axon bundle and RGCs. Retinal axon bundles and RGCs could be recognized by Tuj1 staining. High contrast images were isolated from the immunofluorescence images by FIJI software. **(C)** Quantification of Tuj1-labeled RGCs (^∗^*P* < 0.05, ^∗∗^*P* < 0.01, *n* = 24 fields). **(D)** Quantification of Tuj1-positive axon bundle OD in area of interest (AOI) (^∗^*P* < 0.05, ^∗∗^*P* < 0.01, *n* = 24 fields). **(E)** FG-labeled whole mount retina in sham 7d, sham+LIPUS-7d, ONC-7d, and ONC+LIPUS-7d groups. Scale bar = 500 μm (Enlarged, 50 μm). **(F)** The quantification of FG-positive RGCs (^∗^*P* < 0.05, ^∗∗^*P* < 0.01, *n* = 24 fields).

Retinal ganglion cells in sham, sham+LIPUS7d, and ONC+LIPUS7d groups exerted intact round shape, however, fragmentary shape in ONC7d group (**Figure 2A**). Due to the different density of axon bundles in retina, 1600 μm^2^ in the proximal (*p*) and distal (*d*) regions was chosen in four quadrants from six retinas, respectively. The Tuj1-positive signal was transferred into high contrast signal, and the OD value can be obtained from FIJI quantitatively (**Figure 2B**). As the statistical results indicated, no difference of number of Tuj1-positive RGCs was found between sham and LIPUS-7d groups (**Figure 2C**, ^∗^*P* < 0.05, ^∗∗^*P* < 0.01, *n* = 24 fields). As we expected, the ON crush model significantly decreased the number of Tuj1-positive RGCs and increased the atrophy situation of retinal axon bundles (**Figure 2A**) which is in consistence with the previous data (Lin et al., 2012; Chou et al., 2013). More Tuj1-positive cells could be found in crush+LIPUS group rather than crush group, however, no difference was found between sham and LIPUS groups (**Figure 2C**, ^∗^*P* < 0.05, ^∗∗^*P* < 0.01, *n* = 24 fields). As for the diameters of the axon bundles (width), which can be documented as the progressive degeneration of the RNFL following ON injury using classical neurofibrillary staining methods (Rovere et al., 2015). Crush group (6.833 ± 1.014) has the minimum width compared with sham (16.17 ± 1.222) and crush+LIPUS (17.5 ± 0.992) groups (**Figure 2D**, ^∗^*P* < 0.05, ^∗∗^*P* < 0.01, *n* = 24 fields). Whole-mount retinas were labeled with FG, by confocal *z*-axis scanning and projection, the quantification of FG-positive RGCs could help identify the RGC loss (**Figure 2E**). After ON crush, the number of FG-positive RGCs decreased at 7 d, however, LIPUS treatment prevented the RGC-loss phenotype (**Figure 2F**, ^∗^*P* < 0.05, ^∗∗^*P* < 0.01, *n* = 24). And no difference was found between sham and LIPUS groups (**Figure 2F**, ^∗^*P* < 0.05, ^∗∗^*P* < 0.01, *n* = 24).

### LIPUS Protected Retina From Degenerative Thinning

**FIGURE 3:**
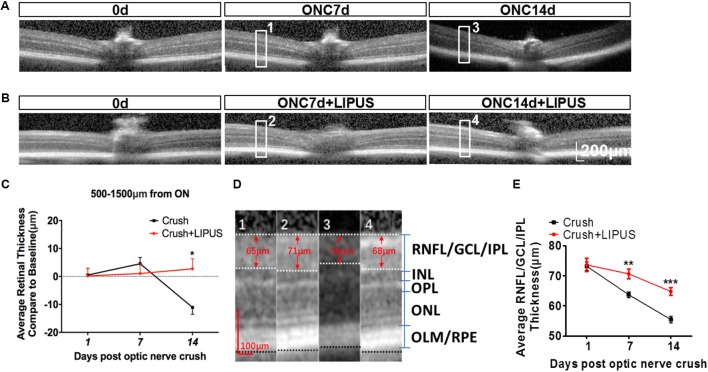
LIPUS protected retina from thinning by OCT examination. **(A)** OCT scanning results of ONC 7 and 14 d retinas in mice. High-power images from ONC0d, 7d, and 14d were obtained from the region of 500–1500 μm from the ON. **(B)** OCT scanning results of ONC0d, 7d, and 14d with LIPUS group. Scale bar = 200 μm. **(C)** The changes of whole retina thickness after the ON crush and LIPUS treatment (^∗^*P* < 0.05, *n* = 7). **(D)** Representative high-power images were obtained from #1, #2, #3, and #4 boxes from ONC 7/14d and ONC 7/14d+LIPUS groups in **Figures 3A,B**. The recognized layers in retina were labeled as RNFL/GCL/IPL, INL, OPL, ONL, and OLM/RPE. Especially, the thickness of RNFL/GCL/IPL was quantified. Scale bar = 100 μm. **(E)** Quantifications of RNFL/GCL/IPL thickness in ONC and ONC+LIPUS groups at 1, 7, and 14 d (^∗∗^*P* < 0.01, ^∗∗∗^*P* < 0.001, *n* = 7). ON, optic nerve; ONC, optic nerve crush; RNFL, retinal nerve fiber layer; GCL, ganglion cell layer; IPL, inner plexiform layer; INL, inner nuclear layer; OPL, outer plexiform layer; ONL, outer nuclear layer; OLM, outer limiting membrane; and RPE, retinal pigment epithelium.

Optical coherence tomography is an ideal method used clinically and experimentally in retinal examination without labeling (Morgan et al., 2017). The resolution approximates that of cellular organelles, which undergo degenerative changes that progress to apoptosis as a result of axon damage. It was showed enlarged images of retina OCT transactions in ONC7d/14d and ONC7d/14d+LIPUS groups (**Figures 3A,B**). The average thickness of the retina was calculated in the area of 500–1500 μm away from the ON disc. In terms of the change of the whole retina thickness compared with the baseline (Δ, vs. baseline) before and after the ON crush from the same animal, the whole retina thickness reduced and the thickness change (Δ) reached statistical significance between the ONC group and LIPUS-treated group at 14 d compared with baseline (**Figures 3A–C**, ^∗^*P* < 0.05, *n* = 7). By different contrast, the retina could be separated into retinal neurofilament layer/ganglion cell layer/inner plexiform layer (RNFL/GCL/IPL), inner nuclear layer (INL), outer plexiform layer (OPL), outer nuclear layer (ONL), and outer limiting membrane/retinal pigment epithelium (OLM/RPE) bands. #1–#4 enlarged images were randomly cropped from ONC7d, ONC7d+LIPUS, ONC14d, and ONC14d+LIPUS retinas, respectively (**Figure 3D**, Scale bar = 100 μm). There were significant decreases of RNFL/GCL/IPL thickness between crush and crush+LIPUS groups at ONC7d and ONC14d, independently (**Figure 3E**, ^∗∗^*P* < 0.01, ^∗∗∗^*P* < 0.001, *n* = 7).

### Grade 1 LIPUS Protected RGCs From Apoptosis After Optic Nerve Crush

**FIGURE 4:**
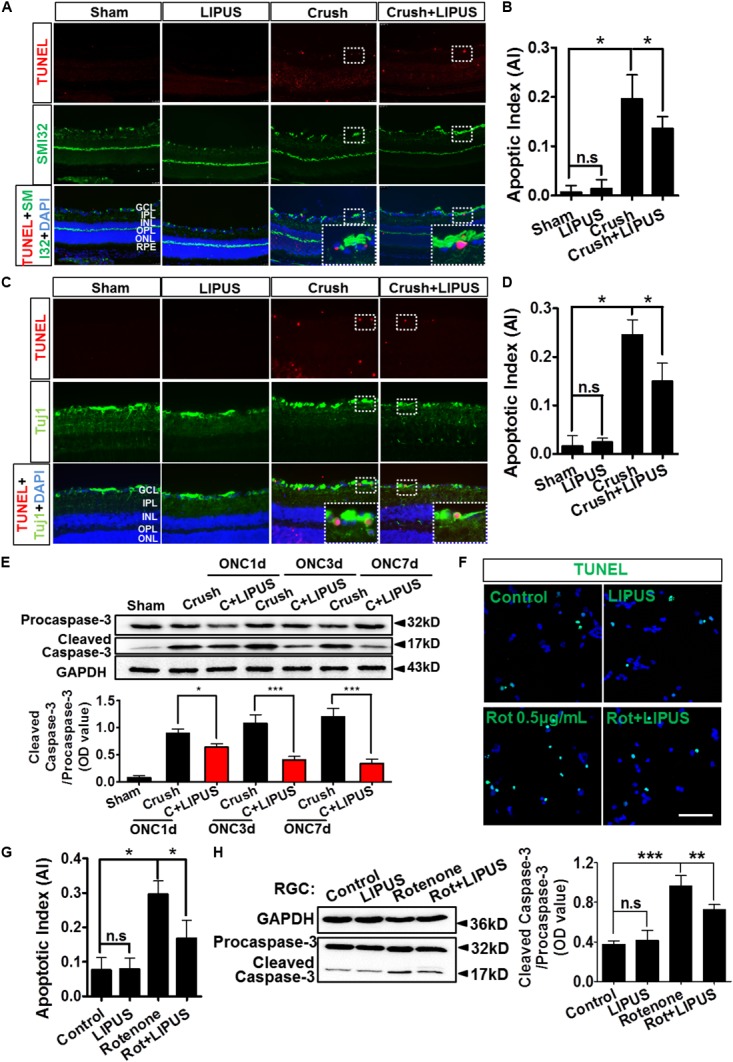
LIPUS protected RGC from apoptosis in ONC model. **(A)** TUNEL staining results (red) in retina sections in sham, LIPUS, crush, and crush+LIPUS groups. TUNEL+ cells were shown in GCL, RGC axon were shown in green by SMI32 immunostaining. High-power image in white boxes showed the co-localization of TUNEL and DAPI in GCL. **(B)** Ratio of apoptotic cells vs. DAPI (*^∗^P* < 0.05, *n* = 6). **(C)** Immunocostaining of TUNEL (red) and Tuj1 (green) to show the apoptotic cells in retina after ON injury. High-power image in white boxes showed the co-localization of TUNEL and DAPI in GCL. **(D)** The quantification of apoptotic cells vs. DAPI (*^∗^P* < 0.05, *n* = 6). **(E)** Western blot detected the expression of procaspase-3 and cleaved-caspase-3 at ONC 1d, ONC 3d, and ONC 7d in crush and crush+LIPUS groups (*^∗^P* < 0.05, *^∗∗∗^P* < 0.001, *n* = 6). **(F)** Primary RGCs were induced by Rot (0.5 μg/ml) *in vitro* injury model. TUNEL staining showed apoptosis of RGCs in control, LIPUS, Rot, and Rot+LIPUS groups. Scale bar = 25 μm. **(G)** Quantification of TUNEL-positive cells vs. DAPI, shown as apoptotic index (AI) (*^∗^P* < 0.05, *n* = 6). **(H)** Western blot results showed more cleaved-caspase-3 expressed in Rot groups. Scale bar = 100 μm (Enlarged, 50 μm). ^∗∗^*P* < 0.01, ^∗∗∗^*P* < 0.001, *n* = 6.

The noninvasive method of ultrasound may have a protective role in RGC apoptosis, however, yet to be defined. As shown in **Figure 4A**, the TUNEL-positive cells were localized mainly in GCL (SMI32-labeled RGC axon). It was quantified that apoptosis is significantly induced by ON crush on the 7th day (crush group), however, less TUNEL-positive cells were observed in GCL in LIPUS group after 7-d treatment (crush+LIPUS group, **Figures 4A,B**, *^∗^P* < 0.05, *n* = 6). To confirm the protective effect of LIPUS on GCL, Tuj1 immunostaining was also performed, which showed similar results as SMI32, more TUNEL-positive cells were found in GCL in crush group (**Figures 4C,D**, *^∗^P* < 0.05, *n* = 6). Procaspase-3/cleaved caspase-3 is a classic apoptotic marker for the mitochondrial RGC apoptosis study. After LIPUS treatment, cleaved caspase-3 in different groups was found decreased in ONC1d, 3d, and 7d, respectively, compared to crush groups (**Figure 4E**, *^∗^P* < 0.05, *^∗∗∗^P* < 0.001, *n* = 4). To confirm the *in vivo* results, primary RGCs were treated with rotenone (Rot), a mitochondria toxin, which could induce RGC mitochondrial apoptosis. After 12 h inducement, more TUNEL-positive cells were found in Rot group rather than Rot+LIPUS groups (**Figures 4F,G**, ^∗^*P* < 0.05, *n* = 6). To confirm the LIPUS protective role on primary RGCs, Western blot results showed increased levels of cleaved caspase-3 in Rot group rather than in Rot+LIPUS group which was in consistence with the TUNEL staining result (**Figure 4H**, ^∗∗^*P* < 0.01, ^∗∗∗^*P* < 0.001, *n* = 6).

### YAP Nuclear Translocation Is Involved in LIPUS Induced RGC Protection

**FIGURE 5:**
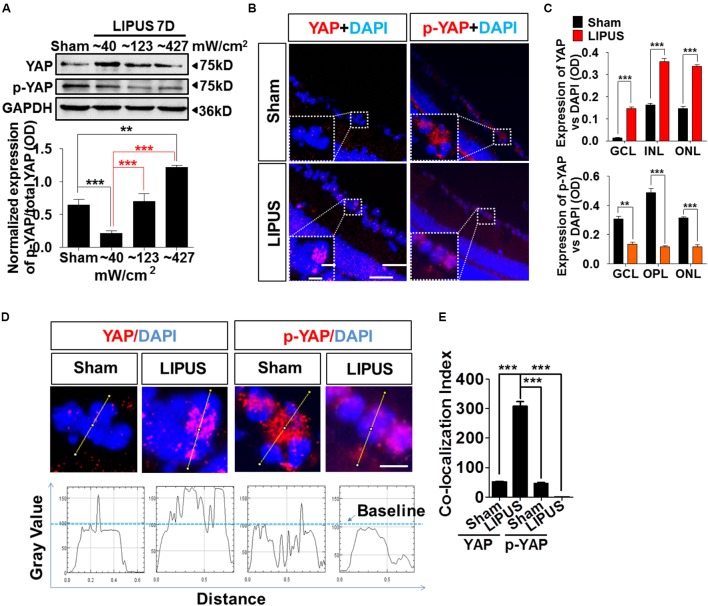
LIPUS promoted YAP nuclear translocation and p-YAP decrease in retina. **(A)** Western blot results showed LIPUS intensity-dependent YAP and p-YAP^S127^ expression at Grades 1, 2, and 5 (^∗∗^*P* < 0.01, ^∗∗∗^*P* < 0.001, *n* = 4). **(B)** Location of YAP and p-YAP^S127^ in retina by IF staining. More YAP-positive cells emerged in RGC, INL, and ONL in LIPUS group. White boxes showed high-power images of relative location of YAP and p-YAP. Scale bar = 100 μm. In-box scale bar = 5 μm. **(C)** Quantifications of YAP and p-YAP^S127^-positive areas in retina vs. DAPI (^∗∗^*P* < 0.01, ^∗∗∗^*P* < 0.001, *n* = 4). **(D)** High-power cellular and nuclear localizations of YAP and p-YAP^S127^. Their line-profiles were generated by FIJI software. OD value in *y*-axis showed the relative intensities beyond baseline. Scale bar = 5 μm. **(E)** Quantifications of co-localization index of YAP/p-YAP with DAPI. ^∗∗∗^*P* < 0.001, *n* = 6.

Yes-associated protein emerged significantly in the set of genes regulated by physical stiffness of ECM and cytoskeletal tension which is required for YAP nuclear localization (Dupont et al., 2011; Dupont, 2016). Increased intensity of LIPUS contributed to the gradually upregulation ratio of p-YAP/YAP (**Figure 5A**, ^∗∗^*P* < 0.01, ^∗∗∗^*P* < 0.001, *n* = 4). YAP upregulated dramatically by Grade 1 (∼40 mW/cm^2^) LIPUS stimulation, but downregulated by Grade 5 (∼427 mW/cm^2^) treatment, which suggested Grade 1 LIPUS is the ideal ultrasound intensity to retina. To localize the YAP and p-YAP pattern before and after the LIPUS treatment, we stained the retina section with specific YAP and p-YAP antibodies. The results indicated that after LIPUS treatment for constitutive 7 d, more YAP-positive puncta were found located in nuclear in GCL (**Figure 5B**, high power field), however, less p-YAP in sham groups which suggested the LIPUS-triggered YAP nucleus translocation and p-YAP inhibition (**Figures 5A,C**, ^∗∗^*P* < 0.01, ^∗∗∗^*P* < 0.001, *n* = 4). YAP was also found localized in nuclear of INL and ONL, increased when LIPUS treated (**Figure 5B**). While p-YAP localized in cytoplasm of RGC (**Figure 5B**, high power field), ONL, and OPL (**Figure 5B**) in sham group, the ODs of YAP-positive puncta were decreased in LIPUS treatment group (**Figure 5C**, ^∗∗^*P* < 0.01, ^∗∗∗^*P* < 0.001, *n* = 6). To distinguish the relative locations of YAP/p-YAP (red) and nuclear (DAPI, blue), we calculated the OD value of their co-localization in the high power inset picture by FIJI software (OD value of YAP-positive signals vs. DAPI-positive signals, **Figure 5D**). Line profile intensity of YAP/p-YAP and nuclear was calculated, the dotted blue line represented a baseline for DAPI (**Figure 5D**). When co-localized, the OD value exceeds the baseline. By statistical calculation, YAP co-localized mainly with DAPI indicating the nuclear translocation of YAP in the LIPUS group. Although the p-YAP expressed much in sham group, less co-localization was examined, which suggested the cytoplasmic localization of p-YAP (**Figure 5E**, ^∗∗∗^*P* < 0.001, *n* = 6).

**FIGURE 6:**
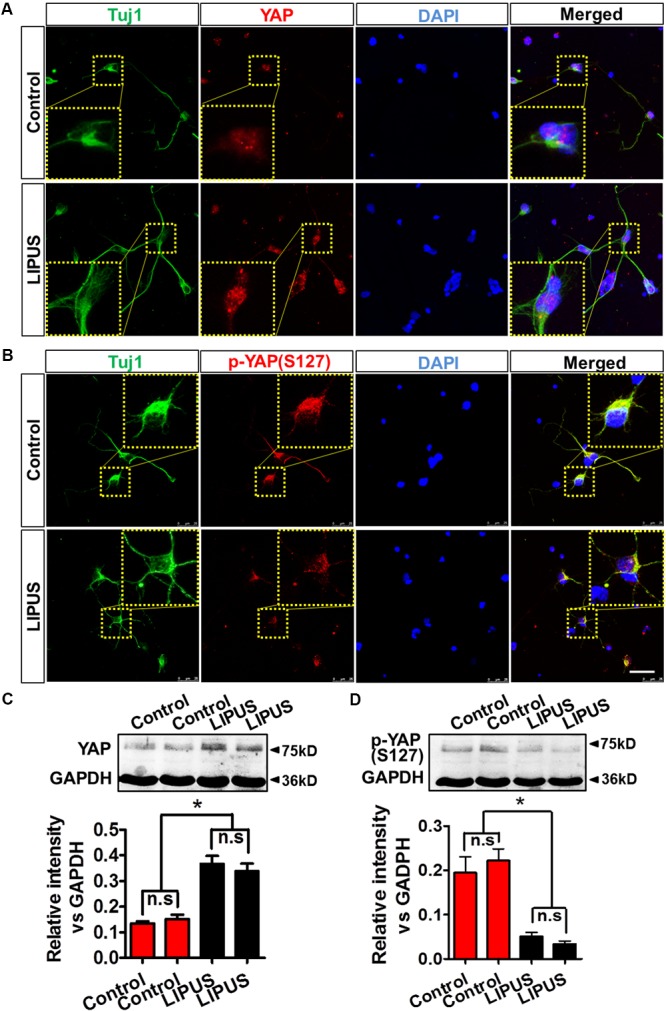
LIPUS promoted YAP nuclear translocation and p-YAP^S127^ decrease in RGCs. **(A)** YAP localization in Tuj1-positive RGCs in control and LIPUS treatment groups. **(B)** p-YAP^S127^ in Tuj1-positive RGCs in control and LIPUS treatment groups. Yellow dotted boxes showed high-power images of relative location of YAP and p-YAP. **(C)** Western blot results of YAP expression of RGCs in control and LIPUS treatment groups. Quantification of OD showed significant difference between control and LIPUS groups. **(D)** Western blot results of p-YAP^S127^ expressions of RGCs in control and LIPUS treatment groups. Quantification of OD showed significant difference between control and LIPUS groups. ^∗^*P* < 0.05, *n* = 6. Scale bar = 5 μm.

As we expected, YAP emerged in the nuclei of primary RGCs (**Figure 6A**). Phosphorylated YAP (Ser127), which stands for the inactivation and cytoplasmic sequestration form of YAP (Zhao et al., 2007; Hao et al., 2008), could also be found in cytoplasm of control and LIPUS-treated primary RGCs (**Figure 6B**). To determine the difference of dynamic pattern of YAP and p-YAP between control and LIPUS groups, we observed the positive signal of transnuclear YAP in both groups and found more p-YAP was dispersed in cytoplasm of primary cortical neuron and primary RGCs in LIPUS treatment groups, however, less YAP was found in control groups (**Figures 6A,B**). To confirm the expression levels of YAP and p-YAP in control and LIPUS treatment groups, primary RGCs were used to perform Western blot after 3-d constitutive LIPUS treatment. In consistence with the IF staining results, the protein levels of YAP increased in the LIPUS treatment group which suggested the power of ultrasound upregulate YAP expression (**Figure 6C**, ^∗^*P* < 0.05, *n* = 6). However, after 3-d LIPUS treatment, p-YAP expression level decreased compared to control groups (**Figures 6B,D**, ^∗^*P* < 0.05, *n* = 6).

### LIPUS Protected RGC From Apoptosis Depending on YAP/Caspase-3 Signaling Pathway

**FIGURE 7:**
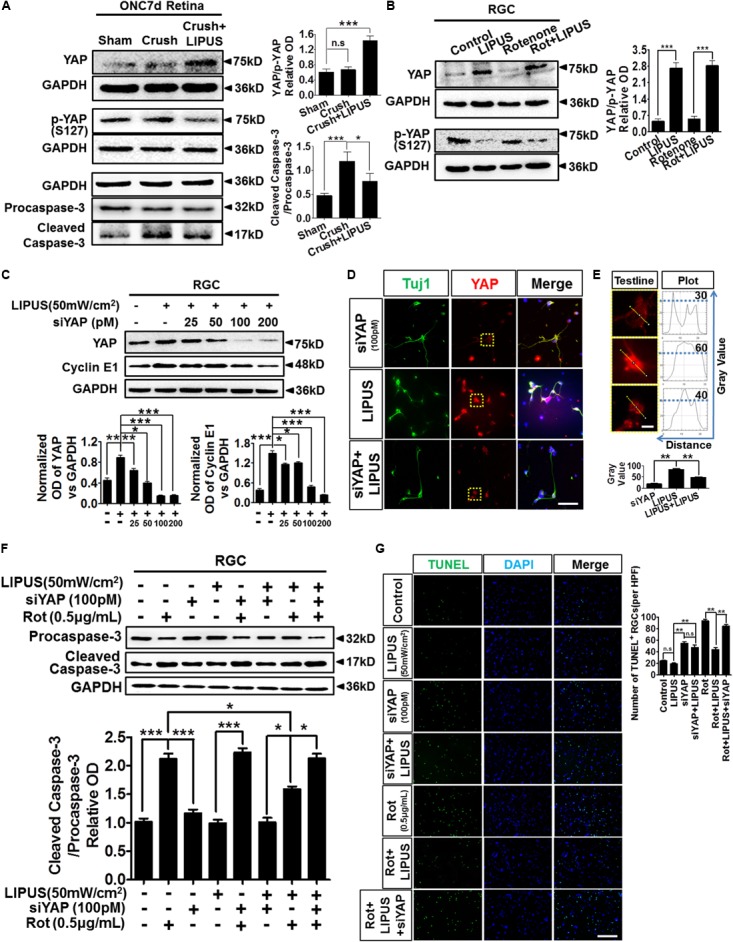
LIPUS protected RGC from apoptosis via YAP-caspase-3 signaling. **(A)** ONC7d retina were dissected and the Western blot detected YAP, p-YAP, caspase-3/cleaved caspase-3 expression levels in sham, crush, and crush+LIPUS groups (^∗^*P* < 0.05, ^∗∗∗^*P* < 0.001). **(B)** Western blot detected YAP, p-YAP, Bcl-2, and Bax expression levels in control, LIPUS, Rot (0.5 μg/ml), and Rot+LIPUS groups in primary RGCs (^∗∗∗^*P* < 0.001, *n* = 4). **(C)** Different concentrations of siYAP (0, 25, 50, 100, and 200 pM) in LIPUS-dependent YAP suppression in RGCs, as well as cyclin E1 expression. GAPDH as internal reference (^∗^*P* < 0.05, ^∗∗^*P* < 0.01, ^∗∗∗^*P* < 0.001, *n* = 4). **(D)** siYAP (100 pM) inhibited LIPUS-dependent YAP (red) expression in Tuj1-positive (green) RGCs. Scale bar = 25 μm. **(E)** The plot profiles of YAP-positive signals in siYAP, LIPUS, and siYAP+LIPUS groups. Presented high-power field of YAP-positive signals origin from yellow-dot boxes in **D**. The curves presented the optic OD value of YAP signal in single RGC origin from yellow test line, which showed highest level of YAP in LIPUS group than other two groups (^∗∗^*P* < 0.01, *n* = 6, scale bar = 5 μm). **(F)** Western blot results showed LIPUS stimulation combines with siYAP and Rot in RGCs. Cleaved caspase-3/procaspase-3 cascade signals were detected in YAP inhibition and Rot-induced neural degeneration condition (^∗^*P* < 0.05, ^∗∗∗^*P* < 0.001, *n* = 4). **(G)** To confirm the Western blot results in **F**, apoptosis of RGCs in different LIPUS stimulation and siYAP inhibition were detect by TUNEL staining. More apoptotic cells were found in Rot group, however, LIPUS partially rescue the Rot-induced RGC neural degeneration (^∗∗^*P* < 0.01, *n* = 6, scale bar = 50 μm).

The ON crush model is widely used for the research of RGC loss and apoptosis, which is initiated by caspase cascade (Berkelaar et al., 1994; Sanchez-Migallon et al., 2016). To further confirm the LIPUS protection in RGC apoptosis after ON crush, paralleled experiments on protein levels of cleaved/caspase-3 were tested. Our data showed comparably increase of cleaved caspase-3 in crush group but slightly decrease in LIPUS treatment group (**Figure 7A**, ^∗^*P* < 0.05, ^∗∗∗^*P* < 0.001). These results indicated that LIPUS protect RGCs from undergoing apoptosis by down-regulating the caspase-3 cascade. In consistent with the YAP and p-YAP immunostaining, higher level of YAP protein was found in the crush+LIPUS group than sham and crush groups, while the expression level of p-YAP was lower than crush group, which demonstrated the vital roles of YAP/p-YAP dynamics in regulating RGC apoptosis, may consider through downstream caspase signaling (**Figure 7A**).

To confirm the molecular mechanism of caspase-3 signaling pathway-induced apoptosis via YAP and p-YAP dynamic behavior, we set up Rot-induced primary RGC injury model and selectively treated with LIPUS. The YAP upregulated in both LIPUS and Rot+LIPUS, while p-YAP downregulated (**Figure 7B**, ^∗∗∗^*P* < 0.001, *n* = 4). To demonstrate the LIPUS promote RGC survival by YAP activation, we suppressed YAP with siYAP in different concentrations from 25 to 200 pM. As the concentration increased, YAP was efficiently knocked down. When YAP was downregulated, as the downstream molecule, Cyclin E1, which controls the proliferation and survival of cell, downregulated depending on siYAP dosage (**Figure 7C**, ^∗^*P* < 0.05, ^∗∗^*P* < 0.01, ^∗∗∗^*P* < 0.001, *n* = 4). The results demonstrated that YAP is the effector of LIPUS stimulation, inhibition of YAP may counteract the ultrasound-triggered cellular proliferation and survival. To verify the expression pattern of YAP in primary RGCs with or without siYAP knockdown, immunostaining of YAP in Tuj1-positive RGCs demonstrated the inhibition role of siYAP in LIPUS-dependent YAP upregulation (**Figure 7D**). Plot profile description of single RGC showed OD of cellular YAP. By quantification, the higher OD value of cellular YAP was found in LIPUS group, however, decreased in siYAP and siYAP+LIPUS groups, which suggested knockdown of YAP inhibits the LIPUS stimulation (**Figure 7E**, ^∗∗^*P* < 0.01, *n* = 6). Next, we adopted LIPUS, siYAP, and/or Rot treatment, respectively, or simultaneously to demonstrate whether the LIPUS contribute protective effects to RGCs survival via YAP-dependent procaspase-3/cleaved caspase-3 cascade signals. As shown in **Figure 7F**, the ratios of cleaved caspase-3/procaspase-3 dramatically increased in Rot and siYAP+Rot groups, however, did not changed in LIPUS and LIPUS+siYAP groups. Furthermore, LIPUS treatment inhibited the Rot-induced-cleaved caspase-3 upregulation to some extent (the 2nd panel vs. the 7th panel, ^∗^*P* < 0.05, *n* = 4). In another word, LIPUS partially rescued Rot-induced RGC apoptosis. Moreover, when siYAP was added to abolish the function of cellular YAP, the cleaved-caspase-3 expression increased dramatically in Rot even under LIPUS treatment, compared to the LIPUS+Rot group (the 7th panel vs. the last panel, ^∗^*P* < 0.05, *n* = 4). Collectively, these results demonstrated that the LIPUS protect RGC from Rot-induced caspase-3-related apoptosis in a YAP-dependent manner (**Figure 7F**, ^∗^*P* < 0.05, ^∗∗∗^*P* < 0.001, *n* = 4). To confirm the apoptosis mechanism, TUNEL staining was used to detect the protective role of LIPUS (**Figure 7G**). As the results, LIPUS reduced the number of TUNEL-positive cells in Rot+LIPUS group compared with Rot group, however, no difference between siYAP and siYAP+LIPUS groups. When siYAP was added to inhibit the LIPUS-dependent YAP upregulation, the number of TUNEL-positive cells increased dramatically compared with Rot+LIPUS group. These results are in consistent with the caspase-3 results (**Figure 7F**). It implies that YAP is the main cellular mechanosensitive factor in protecting RGCs from apoptosis (**Figure 7G**, ^∗∗^*P* < 0.01, *n* = 6). Taken together, these findings indicated that YAP is essential for LIPUS stimulation and pivotal for LIPUS-induced RGC survival by regulating procaspase-3/cleaved caspase-3 signaling.

## Discussion

In contrast to high-intensity ultrasound, LIPUS have the advantages of lower intensity, non-thermo, and non-destruction. In the present study, we have identified the potential effect of LIPUS which was capable of eliciting retinal protection from ON crush-induced RGC apoptosis in a YAP-dependent manner. In support of this conclusion, we found that low grade LIPUS output sustained the cell viability of primary cortical neuron and RGC *in vitro* (**Figures 1A–C**), protected RGC from loss, axon atrophy, and degenerative thinning after ON crush (**Figures 2**, **3**), and protected RGC from ON crush-induced apoptosis and Rot-induced apoptosis *in vitro* (**Figure 4**). For mechanism, we found that the LIPUS can trigger YAP activation, nuclear translocation, and p-YAP inhibition, *in vivo* and *in vitro* (**Figures 5**, **6**), while inhibition of YAP abolished the LIPUS contributed anti-apoptotic effect (**Figure 7**). Together, these results provide novel insight into both molecular and cellular evidences for LIPUS protection of RGCs and could be used to attenuate retinal degeneration.

As previous reported, low-intensity ultrasound did not cause cell damage in the intensity range 100–500 mW/cm^2^ (Lee et al., 2014). In our study, though we utilized the LIPUS device same as Jiang’s which has an acoustic frequency of 1 MHz and 1–20 grades, we clarified the dynamic changes of the acoustic output and provided a whole picture for the following biological effect (Supplementary Figure S1). The time scale and energy output window are pivotal to cell viability. It was reported that lower intensity of LIPUS had better effect than the higher intensity (Jiang et al., 2016). Mechanical energy may contribute to intracellular enzyme activity and metabolism. It was indicated that LIPUS could promote the permeability of cell membrane, enhance mass transfer, and increase absorbing of nutritional elements, however, high-dose LIPUS could result in a high transient intensity of acoustic pressures and high local temperature, which may destroy cell membranes, cytoskeleton, and mitochondria (Kusuyama et al., 2014). Therefore, the ultrasound intensities of 50–500 mW/cm^2^ are widely used as an imaging modality nowadays. Our results indicated that lower energy level (∼40 mW/cm^2^) has beneficial effect on primary RGC and cortical neuron, but higher energy output (∼427 mW/cm^2^) has detrimental effect (**Figure 1E**). In our cell viability experiment, though the maximum treatment duration of LIPUS reach 300 s, we utilized the lowest level of LIPUS output, therefore the temperature of cell dish did not change (**Figure 1I**), which was in consistence with the research of LIPUS biological function in sciatic nerve and mesenchymal progenitor cell lines (Kusuyama et al., 2014; Jiang et al., 2016). Scholtz and Stuhifuth separately confirmed that ultrasound played significant role in clinical effects with nervous tissue (Paprottka et al., 2013). The primary RGCs showed less energy absorption as primary cortical neuron. In mesenchymal stem cells (MSCs), LIPUS suppresses adipogenesis and promotes osteogenesis of MSCs in an energy level of 30 mW/cm^2^. However, in a traumatic brain injury (TBI) model, the LIPUS were applied transracially for a sonication time of 5 min at an acoustic power of 528 mW/cm^2^. These results suggested that different cells and tissue have specific ultrasound energy absorption. And the energy output measurement is necessary for the LIPUS experiments. Our results provided the safe energy-time window for the RGC viability *in vivo* and *in vitro*.

Optic nerve crush is a classic model for research in CNS degeneration, regeneration, and neuronal apoptosis (Levkovitch-Verbin et al., 2000; Li et al., 2016; Sanchez-Migallon et al., 2016). Two to three days after crush injury, distal axonal portions begin to undergo Wallerian degeneration, and 5 d after injury the RGCs undergo apoptosis till nearly 80% RGCs dead in the coming 2 weeks (Park et al., 2008; Hu et al., 2012; Yang et al., 2016) RGCs labeled by retrograde FG injection from superior colliculus/lateral geniculate nucleus (LGN) and whole-mount retinal Tuj1-antibody immunostaining could be observed by confocal microscope, which are common criteria for RGC apoptosis analysis, as we did before (Lin et al., 2012). In the present study, LIPUS treatment did not change the density of the axon bundle in sham group, but protect the NFL from axon loss and axonal atrophy in ONC group, which suggested LIPUS take the function in ON and RGCs protection but not disruption. We provided several lines of evidence to demonstrate the protective role of LIPUS on RGC survival and related axon maintenance, such as Tuj1 immunostaining, FG labeling, TUNEL staining, and OCT examination. This is the first time that clarified the protective role of LIPUS on retinal axon degeneration.

Consideration of axon regeneration, several groups had evidences in the regeneration of the sciatic nerve, which was promoted by LIPUS without temperature changed (Jiang et al., 2016; Zhao et al., 2016). In CNS, although mature axonal regeneration has been focused on for many years and the outcome is promising in *Pten^-^*^/^*^-^* mice (Park et al., 2008), oncomodulin release by inflammatory stimulation from macrophage (Yin et al., 2006), RNA repair (Song et al., 2015), innate immunity (Lin et al., 2012; Baldwin et al., 2015), zinc ion promotion (Li et al., 2017), intrinsic DNA methylation processes (Guo et al., 2011; Weng et al., 2017), and axon growth-promoting cytokines such as CNTF, LIF, and IL-6 stimulation (Leibinger et al., 2013a,b). Seldom has evidence showed non-invasive physical LIPUS-treated retinal axon regeneration. In our work, we have tried to set up the retinal axon regeneration platform, however, 7- and 14-d constitutive LIPUS treatment did not show robust axon regeneration by cholera toxin subunit B (CTB) tracing (data not shown). Due to the limited intrinsic regenerative ability of RGCs, we would like to try to elongate the treatment duration to 1 month or more to investigate the potentiation of retinal axon regeneration in further research work.

Recent studies have indicated the importance of YAP signaling in mediating transcriptional activity from upstream mechanosensitive human Mesenchymal Stem Cell (hMSC) osteogenesis (Xue et al., 2017). Nonetheless, it also indicated that YAP/TAZ has the ability to link cell mechanics to Notch signaling in controlling epidermal stem cell fate and peripheral myelination (Poitelon et al., 2016; Totaro et al., 2017). These evidences suggested YAP/TAZ as the effector of mechanotransduction. We found different levels of YAP changed depending on LIPUS output energy, and the YAP expressed mainly in GCL (**Figures 5B,D**). Nuclear translocation of un-p-YAP is followed by the formation of YAP/TEAD complex that enhances cell proliferation and apoptotic inhibition (Avruch et al., 2012). We also found that LIPUS regulate YAP phosphorylation on serine 127 (Ser^127^) a key YAP1 phosphorylation site residue allows 14-3-3 binding and cytoplasmic sequestration and inactivation (Wang et al., 2009). In our study, YAP and p-YAP^S127^ were both expressed in the retina and RGC when LIPUS treated, however, with opposite cellular behavior, which in consistence with previous results that activation of the Hippo pathway leads to the phosphorylation of YAP at Ser-127, which in turn inhibits the activity of YAP (Basu et al., 2003). To exclude the possible cell-nonautonomous effect, we also verified the effect in isolated and cultured RGCs and found that LIPUS facilitate YAP expression and nuclear translocation, however, alleviated p-YAP expression level (**Figures 5A–C**). In human endothelial cells, laminar shear stress induced YAP translocation (Nakajima et al., 2017), while in oligodendrocytes (OLs), YAP modulated morphogenesis and maturation of OLs in response to mechanical stresses (Shimizu et al., 2017). These evidences indicated that the outer physical stress makes a promising contribution to the activation of some typical cells (Shiu et al., 2018). In our study, except for GCL, YAP and p-YAP were also found expressed in the INL, ONL, and OPL (**Figures 5B,C**). As LIPUS are a type of non-focus ultrasound and have been clinically used as fracture healing treatment from 1994 (Poolman et al., 2017), the acoustic effect on tissue could reach to the deep layer of the retina. Therefore, the LIPUS may trigger the expression pattern of YAP and p-YAP in retina layers other than GCL, which need further elucidation.

Recent studies have indicated that YAP is an anti-apoptotic molecule in podocyte, and podocyte-specific deletion of YAP leads to proteinuria kidney diseases (Rinschen et al., 2017). Moreover, multisite phosphorylation of YAP is crucial and required to protect against apoptosis (Lee and Yonehara, 2012). By LIPUS treatment, either the retina or RGCs were protected by YAP/p-YAP dynamic transformation (**Figures 7A,B**), which strengthened by caspase-3 blotting results. It demonstrated the pivotal role of LIPUS in preventing RGCs from apoptosis via YAP. Nonetheless, YAP could regulate apoptosis by types of phosphorylation (Lee and Yonehara, 2012). In further studies, we would like to test different phosphorylation of YAP in the regulation of retinal degeneration and RGC apoptosis. Multi-evidences have demonstrated that YAP could be activated by LIPUS treatment in this study (**Figures 5–7**). Due to the vital requirement for YAP in the developmental processes of yolk sac vasculogenesis, chorioallantoic attachment, and embryonic axis elongation, homozygosity for the Yap (tm1Smil) allele (*Yap^-^*^/^*^-^*) caused developmental arrest around E8.5 (Morin-Kensicki et al., 2006). Therefore, *Yap^-^*^/^*^-^* mice cannot be suitable for further research work. Cyclin E1 is the potential downstream effector of YAP which contributes to cell cycle and cell growth (Huang et al., 2005; Corvaisier et al., 2016) and the downregulation of it may involve in cellular apoptosis (Gurzov and Izquierdo, 2006). The suppression of Cyclin E1 depended on the gradual concentration of siYAP suggested YAP is the predominant molecule that mediates LIPUS-triggered potential cell viability (**Figure 7C**). Although not all Cyclin E1 decreased with YAP knock down, other signaling pathway may be involved in Cyclin E1 activation. To confirm the pivotal role of YAP in LIPUS mechanotransduction and anti-apoptotic effect, procaspase-3/cleaved caspase-3 cascades were detected, which are typical cellular mitochondria injury markers. As we found, the LIPUS protected RGCs from mitochondrial injury without YAP knock down (**Figures 7F,G**), thus which suggested YAP is the mediator of LIPUS and apoptosis. In sum, the results indicated that the LIPUS contributed to RGC survival and protect RGC from apoptosis by YAP activation, nuclear translocation, caspase-3 cleave, and even cyclin E1 activation. It would be very interesting to explore the potential upstream and downstream signaling pathways that could trigger YAP activation, phosphorylation, and upcoming cellular effects.

## Author Contributions

SL designed the study and wrote the manuscript. JY provided the funding and helped discuss the study. J-XZ, XC, and Y-JL performed the experiments and analyzed the data. XZ and JX helped in OCT measurement and immunostaining. KY and DW helped in LIPUS adjustment, procurement, and facilitated the ultrasound generator. All authors read and approved the final manuscript.

## Conflict of Interest Statement

The authors declare that the research was conducted in the absence of any commercial or financial relationships that could be construed as a potential conflict of interest.
