# Supplementary material for: Low-Intensity Pulsed Ultrasound Protects Retinal Ganglion Cell From Optic Nerve Injury Induced Apoptosis via Yes Associated Protein
Source: Front Cell Neurosci. 2018 Jun 13;12:160. doi: 10.3389/fncel.2018.00160 (PMC6008403; doi:10.3389/fncel.2018.00160)
Supplement: Supplementary file 1 [file Image_1.PDF]

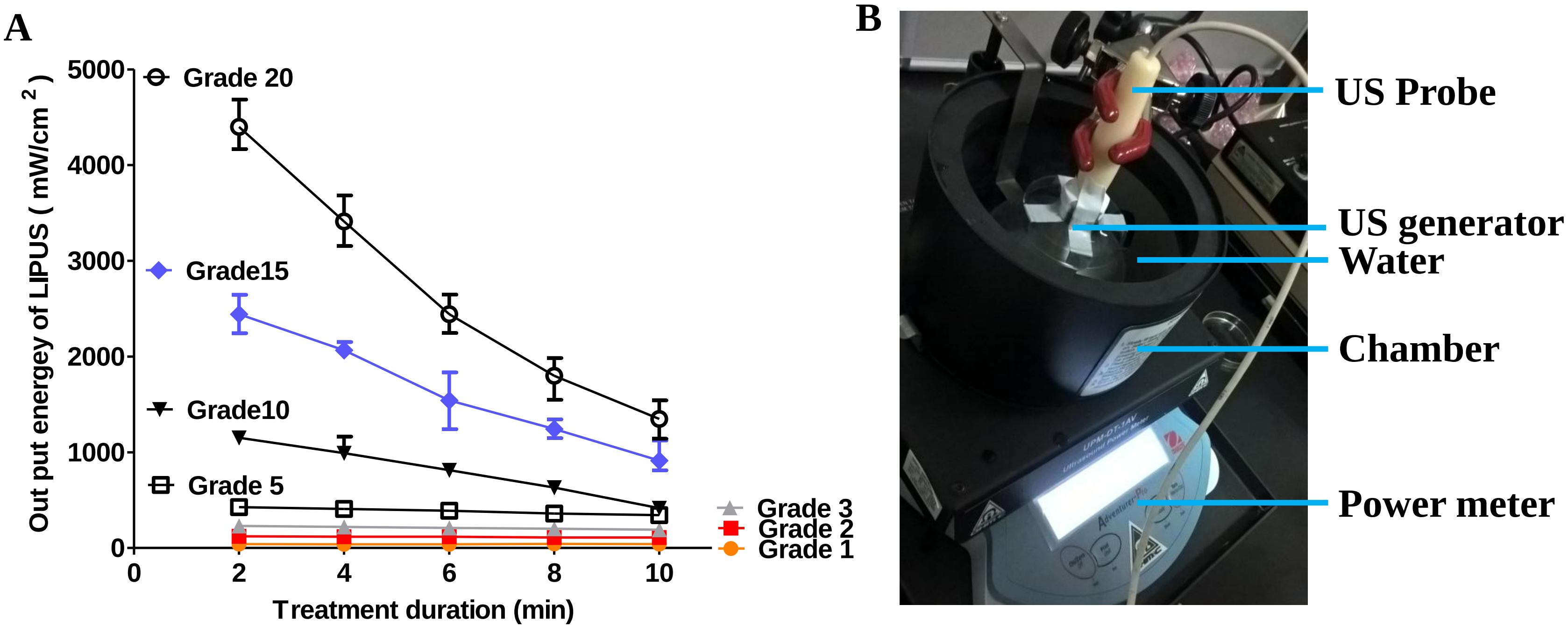

Figure S1. Ultrasound energy gradesustained stable at Grade1 and Grade2 energy grades but unstable at Grade3, Grade5, Grade10, Grade15 and Grade20 grades

Referred to the literature, stimulation in CNS ranged from 30mW/cm^2 to 100mW/cm^2 below the recommended limit for clinical diagnostic imaging application. However, the energy and intensity were varied depending on different apparatus and experiments, which ranged from 1.0 to 2.5MHz and the intensities of 0.1-1.5W/cm^2. As we tested, the energy in Grade1 grade ranged between 40.5±1.857 and 40.3±0.919 mW/cm^2 from 2nd to 10th min measurement, Grade2 ranged between 123±2.781 and 110.667±3.138 mW/cm^2, and Grade3 ranged from 230.333±2.985 to 191±1.915 mW/cm^2. As for high power output, Grade5 ranged from 427.167±8.345 to 354±10.467 mW/cm^2, Grade10, Grade15 and Grade20 grades range from 1151.833±9.393 mW/cm^2 to 416.333±17.175 mW/cm^2, 2441.833±65.393 mW/cm^2 to 914.167±46.064 mW/cm^2 and 4400.5±84.219 mW/cm^2 to 1348.5±55.253 mW/cm^2, respectively. However, the energy of the three high grades unstable after 2min-test and attenuated gradually (Table 1).

Ultrasound energy measurement

LIPUS is now becoming a promising clinical treatment for bone healing [22], and a potential physical stimulation form and therapeutic approach for PNS axon outgrowth [23, 24]. However, the precise energy outputs of LIPUS were not consistent between different ultrasound generators. The measurement of US energy (Ultrasonic probe diameter: 2.5 cm, acoustic frequency 1 MHz, duty cycle: 20%, pulsed repetition frequency 1 KHz. Ultrasonic therapeutic apparatus was designed and manufactured by medical ultrasound engineering institute of Chongqing medical university, China) is thus important for the coming experiments in vitro and in vivo. We utilized ultrasound power meter (UPM-DT-1AV, Ohmic Instruments, US) to measure the ultrasound output energy and consistent level. Briefly, the ultrasonic probe was gripped by the gripper and the metal part of the generator was placed under water above the detect cone. We chose different power levels to measure the output acoustic intensity, from Grade1 (minimum) to Grade 20 (max). Each grade repeated for independent six times, and the outputs of energy were showed as Joule (J, mean ± S.E.M.).
